# Comparison of methods for identifying the optimal treatment duration in randomized trials for antibiotics

**DOI:** 10.1186/s13063-025-09050-y

**Published:** 2025-09-24

**Authors:** Suzanne M. Dufault, Brian H. Aldana, Patrick P. J. Phillips

**Affiliations:** 1 Division of Biostatistics, Department of Epidemiology and Biostatistics, University of California, San Francisco, CA USA; 2 UCSF Center for Tuberculosis, San Francisco, CA USA; 3 Division of Pulmonary and Critical Care Medicine, Department of Medicine, University of California, San Francisco, CA USA

**Keywords:** Tuberculosis, MCP-Mod, Duration-ranging, Antibiotics

## Abstract

**Background:**

The optimal duration of antibiotic treatment must strike a delicate balance: it must be long enough to achieve desirable efficacy yet short enough to prevent the development of toxicities, adverse events, and mitigate other arduous aspects related to patient burden. Historically, the approach used to determine duration of antibiotic treatment has been inefficient, severely impacting the refinement of therapeutics for tuberculosis (TB) where treatment duration, and its complications, can be extensive. Many of the challenges in duration-ranging have parallels and proposed solutions in the field of dose-ranging where the literature is substantially more established and where the traditions of qualitative, pairwise comparison studies have been replaced with model-based approaches. Such methods are more efficient and allow for interpolation between the doses observed.

**Methods:**

This work examines the utility of cutting-edge dose-finding methods (such as MCP-Mod) for duration-ranging of TB treatments. We compare the operating characteristics of the adapted model-based duration-ranging methodologies against standard qualitative methods for the purposes of estimating optimal duration and describing the duration-response relationship, using a simulation study motivated by a Multi-Arm Multi-Stage Response Over Continuous Intervention (MAMS-ROCI) clinical trial design. We explore three specific targets: (1) power to detect a duration-response relationship, (2) ability to accurately reproduce the duration-response curve, and (3) ability to estimate the optimal duration within an acceptable margin of error.

**Results:**

We find that model-based methods outperform standard qualitative comparisons on every target examined, particularly when the sample size is constrained to that of a typical Phase II trial.

**Conclusions:**

We conclude that the success of the next era in TB therapeutics duration evaluation trials, and antibiotics duration-ranging more broadly, will meaningfully rely on the ability to simultaneously pair innovative model-based statistical methods with re-imagined study designs such as MAMS-ROCI.

**Supplementary Information:**

The online version contains supplementary material available at 10.1186/s13063-025-09050-y.

## Background

Tuberculosis (TB) is a complex infectious disease requiring 6 months of multi-drug treatment with four drugs to effect cure; longer if the disease has resistance to key first-line drugs [1]. An estimated 10.6 million people developed TB in 2022, of whom only two thirds were diagnosed and started on treatment [1]. While the success rate hovers around 86% [2] when taken as intended, the many challenges associated with the arduous duration of treatment (e.g., toxicities, adverse events, patient burden) underscore the need for shorter, safer regimens [2–4] and many new drugs are in clinical development [5]. Duration of any new regimen is a critical yet delicate balance: it must be long enough to achieve desirable efficacy yet short enough to alleviate the aforementioned challenges.

Evaluating the optimal duration of a new regimen adds complexity to clinical trials that must also identify the most favorable combinations of drugs at the proper safe and effective doses. Trials for new TB regimens have therefore typically evaluated only one or, at best, a narrow range of few durations within a single trial, comparing each shortened duration against a common control [6]. Pairwise comparison of different treatment durations is not an efficient mechanism to determine the optimal duration, nor does it directly allow the exploration of intermediate durations that are not observed within the trial. The Rapid Evaluation of Moxifloxacin in the Treatment of Sputum Smear Positive Tuberculosis Trial (REMoxTB) trial found two 4-month regimens were not non-inferior to the 6-month control [7], although mouse studies have since suggested that if one had been given for 5 months it probably would have achieved non-inferiority [8]. Bedaquiline was only studied for 6 months in clinical trials [9], but has since been show to be effective in longer regimens and WHO guidelines have included regimens with at least 9 months of bedaquiline [10].

Many of the challenges in identifying an optimal duration have parallels and proposed solutions in trials seeking to identify an optimal dose, where the Literature is substantially more established. Prior to the 1990s, dose-ranging studies approached dose as a qualitative nominal variable, evaluating the evidence for dose-response relationships using multiple comparison methods such as ANOVA and variations on Dunnett’s method [11]. Such approaches do not permit interpolation of the dose-response relationship to dosages that were not studied and tend to overestimate the minimum effective dose. Approximately 10% of all drugs licensed by the USA Food and Drug Administration (FDA) in the 1980s were recently re-dosed with most dosages dropped nearly 33% [11]. In the last few decades, model-based approaches for dose-finding have become more common. These methods treat observed dosages as observations on a continuous variable [12], are more efficient requiring fewer participants, and allow for interpolation between the doses studied. Pre-specification of the corresponding dose-response curve is critical; some advocate the use of a fully flexible model (e.g., fractional polynomials, splines, nonparametric models) [13], others a more restrictive monotonic flexible model (e.g., sigmoid Emax [14]), and still others the prespecification of a candidate set of parametric models and a procedure for either model selection or model averaging [15]. This last approach, referred to as MCP-Mod, is a dose-finding method that combines the principles of multiple comparison procedures (“MCP”) with modeling (“Mod”). It has been shown to address the limitations of either approach performed in isolation and has been qualified by the FDA (2016) and the European Medicines Agency (EMA) (2014) as an “adequate and appropriate method for dose selection” [16, 17]. The objective of our work was to adapt and evaluate these model-based methods for duration estimation.

## Methods

The methods for this simulation study are organized according to the “Aims, Data-generating mechanisms, Methods, Estimands, Performance Measures” (ADEMP) framework proposed by Morris et al. [18].

### Aims

Two aims motivate this work. First, we aimed to adapt candidate model-based dose-ranging methodologies for the task of duration-ranging. Second, we aimed to compare through simulation study the operating characteristics of model-based duration-ranging methodologies against standard qualitative methods to identify the preferred method for estimating the optimal duration and for describing the duration-response relationship.

### Data-generating mechanism

**Fig. 1:**
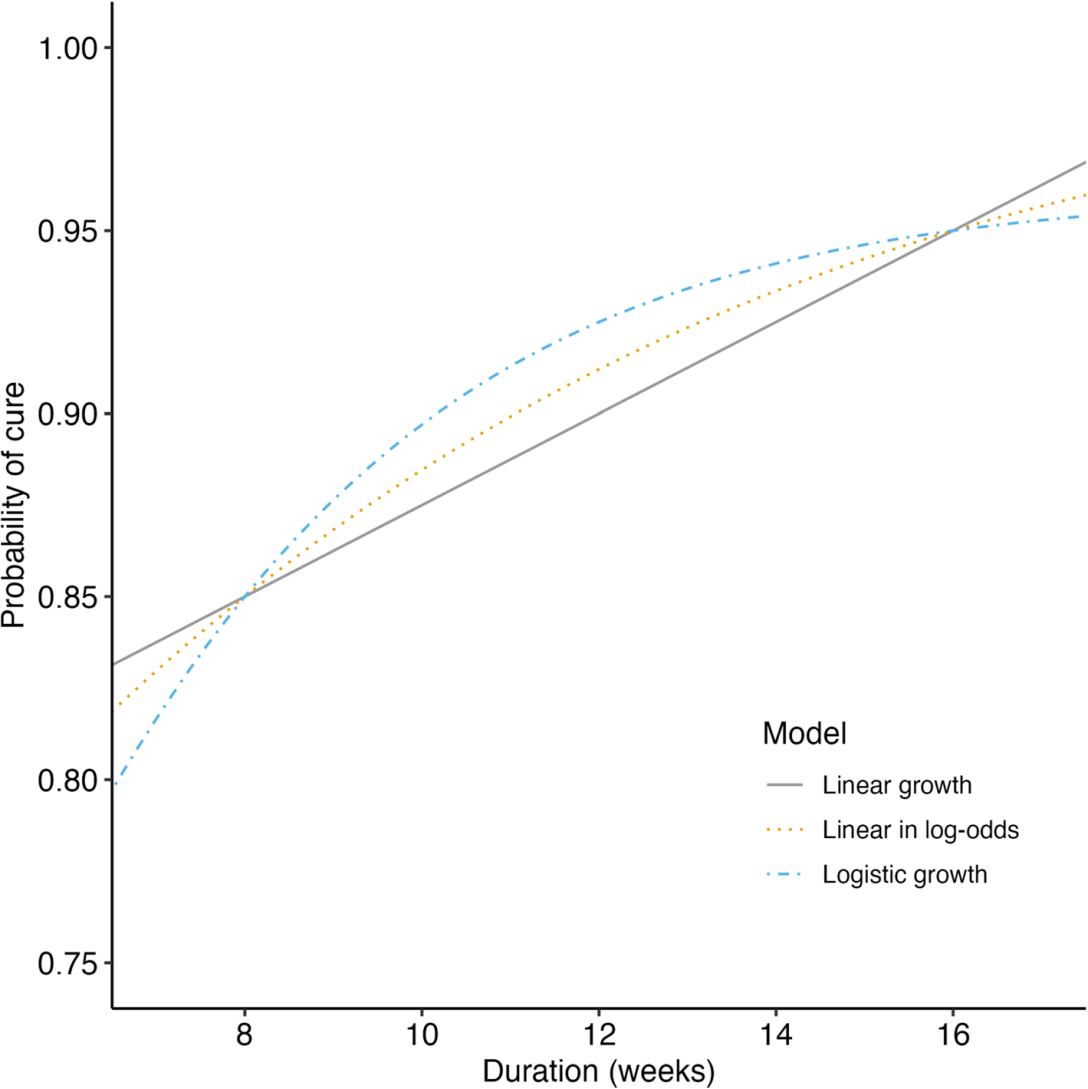
Data were generated according to parametric models. All models were parameterized such that the probability of cure given 8 and 16 weeks of treatment is 0.85 ($\mathbb {E}[Y|X=8] = 0.85$) and 0.95 ($\mathbb {E}[Y|X=16] = 0.95$), respectively. Exact parameter values are included in the Appendix (Table S1)

We randomly assigned participants to the exposure of interest, *X*, duration of treatment, ranging from 8 to 16 weeks, evenly allocated across 2-week increments, reflecting the range of interest for drug-sensitive TB (DS-TB) therapeutics trials. The outcome, *Y*, was simulated as a binary endpoint defined as the presence of an unfavorable event (treatment failure or relapse) by the end of follow-up at 52 weeks post-randomization. The binary outcomes were simulated as a function of treatment duration ($Y\sim f(X)$) according to a range of pre-specified data-generating mechanisms shown in Fig. 1 (linear, log-linear, and logistic). All models were parameterized such that the probability of cure given 8 and 16 weeks of treatment is 0.85 ($\mathbb {E}[Y|X=8] = 0.85$) and 0.95 ($\mathbb {E}[Y|X=16] = 0.95$), respectively, and the expected “true” response rate at any given duration can be derived exactly from the parameterized relationship. Exact parameterizations are included in the Appendix (Table S1), and motivated by the expected efficacy of current DS-TB multidrug regimens [7].

### Estimands

The statistical targets ($\varvec{\psi } = \{\psi _1, \psi _2, \psi _3\}$) correspond to the duration-adapted motivating questions in Table 1. First, we target whether there is evidence of an effect, $\psi _1$. The second target, $\psi _2$, is the estimated duration-response curve, and $\psi _3$ is the estimated optimal duration, defined in this work as the minimum duration associated with a pre-specified “acceptable” relapse rate, specifically a relapse rate of 90%. We will refer to this as the minimum effective duration (MED).

**Table 1:** Motivating questions and statistical approaches – standard qualitative versus adapted model-based – used to estimate the corresponding targets

Question	Statistical target	Standard qualitative approach	Adapted model-based approach
Is there evidence of an effect?	$\psi _1$	Test of PoC: ANOVA	Test of non-zero slopes in candidate models
What is the duration-response relationship?	$\psi _2$	–	Fitting of duration-response curve using candidate models
What is the optimal duration (weeks)?	$\psi _3$	Pairwise one-sided tests of equivalence or non-inferiority, adjusted for multiple testing as appropriate	Direct estimation of optimal duration

Note: optimal may be defined in many ways and the proposed approaches may not be appropriate for all definitions. For this work, we define optimal as the minimum duration associated with a pre-specified relapse rate

### Methods of analysis

The statistical targets ($\varvec{\psi }$) were estimated using model-based and standard qualitative (pair-wise comparison) methods listed here (Table 2). The standard qualitative assessment framework does not directly empower estimation of the duration-response curve ($\psi _2$), so only the model-based methods were compared on this target.

**Table 2:** Methods of analysis, their categorization as either novel model-based or standard qualitative, and the statistical targets (estimands) they can estimate

Method	Categorization	Targets Estimated
MCP-Mod (select)	Model-based	$\psi _1,\psi _2,\psi _3$
MCP-Mod (average)	Model-based	$\psi _1,\psi _2,\psi _3$
Fixed 1-degree fractional polynomial	Model-based	$\psi _1,\psi _2,\psi _3$
Fixed 2-degree fractional polynomial	Model-based	$\psi _1,\psi _2,\psi _3$
Linear splines	Model-based	$\psi _1,\psi _2,\psi _3$
Dunnett test	Qualitative	$\psi _1,\psi _3$

**MCP-Mod:** MCP-Mod can be utilized in either a model selection or model averaging framework; both were evaluated here. For both procedures, the candidate model library included the following models standard in dose-ranging, with parameterizations (Table S2) and visualizations (Fig S1) included in the Supplemental Material: Emax, sigmoid Emax, quadratic, and linear [19]. For model selection (“MCP-Mod (select)”), all candidate models in the candidate model library were fit. If at least one of the candidate models detected a significant relationship between duration and response after adjustment for multiplicity [15], this was taken as evidence of effect ($\psi _1$). The model with the minimum Akaike Information Criteria was selected, with information criteria penalized as previously defined and henceforth referred to as gAIC (MCP-step) [12, 20]. This model was then used to estimate the duration-response curve ($\psi _2$) and identify the MED ($\psi _3$), defined as the minimum duration corresponding to an estimated mean response rate greater than or equal to 90% (Mod-step). For model averaging (“MCP-Mod (average)”), the MCP-step ($\psi _1$) was the same. The Mod-step averaged the model fits from the best performing models across 100 bootstrap resamples of the data [12] in order to estimate the duration-response curve ($\psi _2$), with the MED ($\psi _3$) again defined as the minimum duration associated with a bootstrapped median response rate greater than or equal to 90%. The median of the bootstrapped response rates was chosen to be robust to outliers.

**Fractional polynomials:** Fixed 1- and 2-degree fractional polynomials were fit (“FP1” and “FP2,” respectively). Evidence of effect ($\psi _1$) was determined when the gAIC of the fractional polynomial model was lower than the gAIC of an intercept only model. The fitted models were then used to estimate the duration-response curve ($\psi _2$) and to identify the minimum duration ($\psi _3$) corresponding to an estimated mean response rate greater than or equal to a 90% response rate.

**Linear splines:** Piece-wise Linear splines with 2 knots were fit under two conditions: with equally spaced knots (LS2e) and with 2 knots manually set (LS2m) at *k* = 10 and 12 weeks, as described in [4]. In both applications, evidence of effect was determined by a statistically significant, non-zero slope ($\psi _1$). The fitted models were then used to estimate the duration-response curve ($\psi _2$) and to identify the MED ($\psi _3$) corresponding to an estimated mean response rate greater than or equal to 90%.

**Dunnett test:** A standard qualitative method for dose-ranging, we performed Dunnett’s (1955) multiple comparisons procedure to compare the response rates among the discrete durations, using the longest duration as the control. Evidence of effect ($\psi _1$) was established if there was at least one duration at which the observed relapse rate was estimated to be different from the longest duration’s relapse rate, at a statistically significant level ($P < 0.05$) where the *p*-value has been adjusted to account for multiple comparisons as described in [11]. Unlike the model-based methods, we are unable to directly identify the duration likely corresponding to a particular response rate. Instead, duration estimation is performed by comparing the performance of each shortened duration relative to the longest duration, which is expected to have the highest proportion of cure. The MED ($\psi _3$) was then determined one of two ways. First, we implemented a closed testing approach, using a step-wise procedure similar to that described in [11]. Beginning with the comparison between the two longest durations, the estimated 90% simultaneous confidence bound on the difference in proportion of cure was compared to a clinically meaningful difference of 0.06. If the lower confidence bound was within this margin, the search for the optimal duration continued by comparing the results from the third longest duration versus those from the longest. This procedure continued until the lower confidence bound exceeded the margin, at which point the shorter duration had exceeded a clinically meaningful decline in performance relative to the longest duration available, and the penultimate duration evaluated was considered to be the MED. Second, we implemented an open testing approach, performed as an unordered procedure also described in [11], in which the MED ($\psi _3$) was assumed to be the minimum duration whose 90% simultaneous confidence interval was estimated to fall below the clinically meaningful difference of 0.06, agnostic to any discontiguous duration-response intervals.

Exact parameterizations of each method can be found in the Supplemental Material.

### Performance measures

**Power:** Assuming each of the data-generating mechanisms were equally likely, the estimators’ performance in detecting evidence of effect ($\psi _1$) was compared based on the proportion of simulated datasets across which there was sufficient evidence detected to reject the null hypothesis of a flat duration-response curve. Specifics corresponding to each method are included in the “Methods of analysis” section.

**Scaled area between curves:** The scaled area between the estimated and true curves [4] was used to reflect estimator performance in capturing the true duration-response curve ($\psi _2$). This metric averaged the absolute model performance across the entire curve and was scaled such that the possible values range from 0 to 1, where 0 is a perfect fit. Qualitative approaches cannot estimate the duration-response curve ($\psi _2$), and therefore performance on this target was only compared among the modeling methods.

**POS**_**MED**_**:** While there were three targets of interest, priority was given to the estimation of the optimal duration ($\psi _3$), defined as the minimum duration associated with a relapse rate of 90%, or MED. The statistical performance of each estimation method in this regard was measured as the probability of successfully estimating the MED within a certain range (POS_MED_) [21]. This performance metric accounts for both estimator bias (i.e., accuracy in estimating the true optimal duration) and variance (i.e., the expected precision associated with the estimator) when evaluating estimator performance.

We examined POS_MED_ under three conditions, outlined in Eq. 1–3. First, we examined the probability that the estimated MED fell within the interval between the true MED and the maximum duration observed (Eq. 1). This is aligned with what Quartagno et al. [22] refer to as the “acceptable power.” Next, we examined the probability that the estimated minimum duration fell within two intervals around the true minimum where both intervals assume it is better to err on selecting a duration that is slightly too long than one that is too short. We examined the probability that the estimated MED was no shorter than the truth by 1 (Eq. 2) or 2 weeks (Eq. 3), while also being no more than 2 (Eq. 2) or 4 (Eq. 3) weeks longer than the truth. As an example, if the true optimal duration ($\psi _3$) is 9 weeks, Eq. 2 would accept as “successful” any method with an estimated duration ($\widehat{\psi _3}$) ranging between 8 and 11 weeks while Eq. 3 would expand the definition of success to any estimated duration ($\widehat{\psi _3}$) ranging between 7 and 13 weeks.

1 $$\begin{aligned} \text {POS}_{\text {MED}, [0,\max )} & = \Pr \left( \widehat{\psi _3} \in [\psi _3, \max (d))\right) \end{aligned}$$

2 $$\begin{aligned} \text {POS}_{\text {MED}, [-1,+2]} & = \Pr \left( \widehat{\psi _3} \in [\psi _3-1, \psi _3+2]\right) \end{aligned}$$

3 $$\begin{aligned} \text {POS}_{\text {MED}, [-2,+4]} & = \Pr \left( \widehat{\psi _3} \in [\psi _3-2, \psi _3+4]\right) \end{aligned}$$

The performance of all three metrics for varying sample sizes was used to explore estimator robustness.

All code necessary for data generation, analysis, and report compilation is publicly available in a GitHub repository maintained by the first author (https://github.com/sdufault15/tb-mcp-mod). All analyses were performed in R version R 4.4.1 (2024-06-14) “Race for Your Life,” using “DoseFinding” [23] and “MCPMod” [19] packages for performing the MCP-Mod analyses, “lspline” [24] for the linear splines, “gamlss” [25] for the fractional polynomials, “DescTools” [26] for the Dunnett Tests, “tidyverse” [27], and “patchwork” [28] for data wrangling and figure generation.

## Results

### Method performance overall

A high-level summary of each method’s performance across various metrics is shown in Table 3. These results are compiled for 15,000 simulated datasets at each sample size, assuming each data-generating mechanism is equally likely. Performance across each individual data-generating mechanism is explored in the subsequent subsections.

**Table 3:**
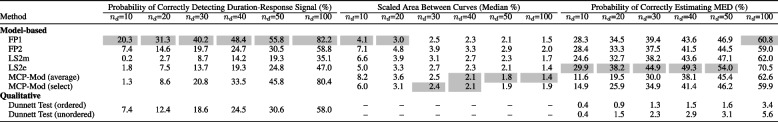
Probability of correctly detecting a duration-response signal (power), the scaled area between the true duration-response curve and the estimated duration response curve by sample size for each estimation method, and the probability the estimated minimum effective duration (MED) falls within a reasonable range around the true MED ($\text {POS}_{\text {MED},[-1,+2]}$)

The best performing method at each sample size for each metric is highlighted in gray

The power to detect evidence of effect ($\psi _1$) is captured in the first set of columns. As expected, across each method, power increases as the sample size randomized to each duration ($n_d$) increases. Note, the two MCP-Mod approaches apply the same method to detect evidence of effect (MCP-step), and therefore, their results are identical and pooled in Table 3. The same is true for the two Dunnett test approaches; their results have been similarly pooled. Fractional polynomials have the best power to detect a duration-response signal, with 80% power with 100 participants per duration ($n_{total} = 500$). The other methods, model-based and Dunnett, require approximately two to three times as many participants to achieve equivalent power to FP at lower sample sizes.

The method-specific performance in terms of accurately estimating the underlying duration-response curve ($\psi _2$) summarized via the sABC metric is captured in the second set of columns. Here, the MCP-Mod methods have the best performance with only 2.4% average absolute error in estimating the probability of cure across the range of durations observed when there are 30 participants randomized to each duration ($n_{total} = 150$). FP1 is similarly competitive.

Method-specific performance in accurately estimating the MED ($\psi _3$) within a reasonable range around the truth is captured in the third set of columns. These columns capture the proportion of analyses where the estimated MED fell within an interval of no less than one week below the true MED and no more than 2 weeks greater than the true MED ($\text {POS}_{\text {MED},[-1,+2]}$). Given the relative Linearity of the underlying DGMs, it is perhaps unsurprising that the Linear spline methods appear to have best performance on this metric, despite having poor overall fit of the duration-response curve. The Dunnett Test has the poorest performance, consistently failing to select any duration lower than 16 weeks (Supplemental Figure S2).

### Performance: evidence of effect

**Fig. 2:**
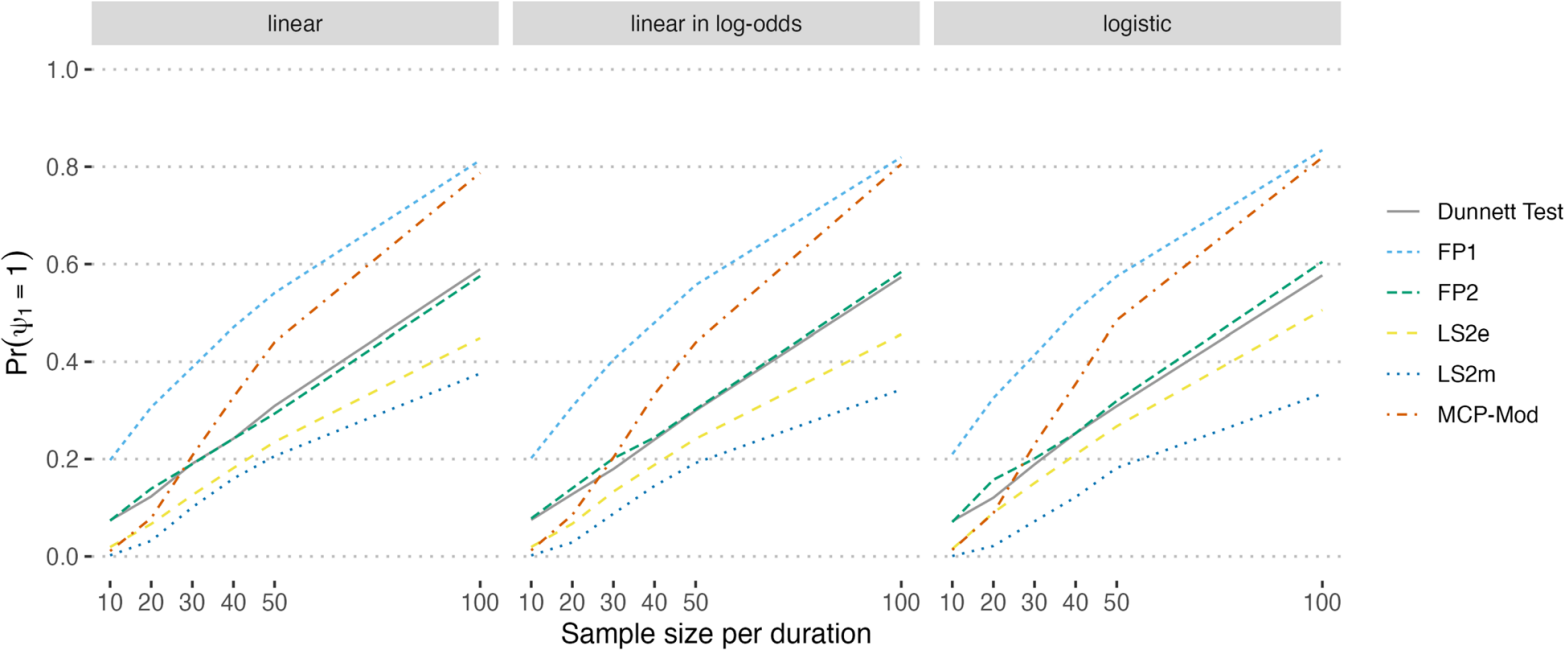
The proportion of simulated datasets across which there was sufficient evidence detected to reject the null hypothesis of a flat duration-response curve (power)

The methods perform quite similarly in terms of power across all data-generating mechanisms, with slightly improved performance observed in the logistic growth setting (Fig. 2: logistic). The FP1 and MCP-Mod methods achieved at least 80% power when 100 participants were randomized to each duration. Across sample sizes less than 100 per duration, the estimated power was lower than would be acceptable in a classic Phase III study, but may be sufficient for an exploratory Phase II study. For example, the maximum probability of detecting evidence of an effect only reaches 58.8%, even at a sample size of 50 per duration (Fig. 2). The power to detect an effect for the Dunnett Test is not notably different than the other model-based methods and has the second highest power when the sample size is extremely small ($n_d = 10$).

### Performance: duration-response curve fit

**Fig. 3:**
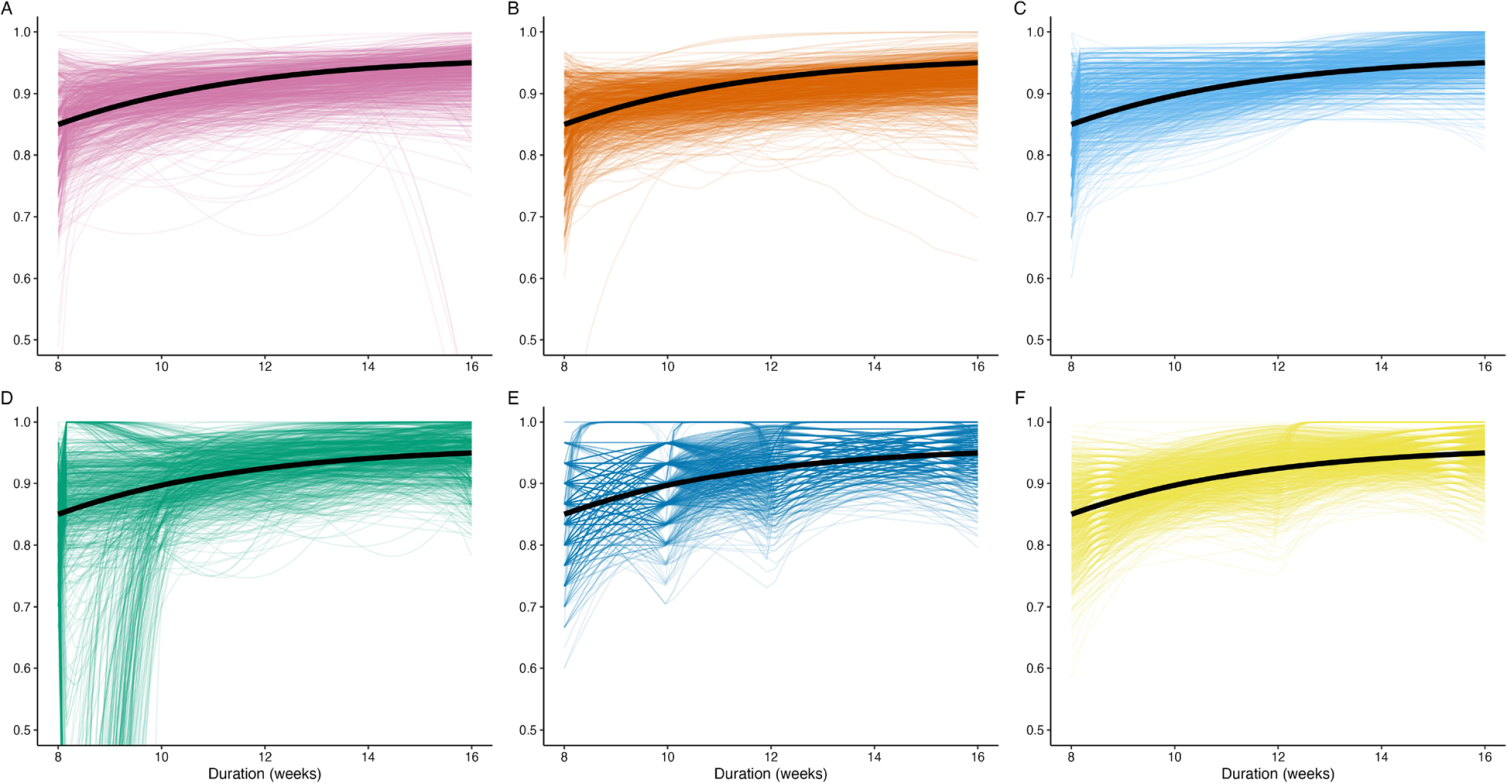
Estimated duration-response curves from 1,000 datasets simulated from the logistic data-generating mechanism plotted against the true duration-response curve (black) for a sample size of 30 participants per duration, evenly distributed across the five durations. **A** MCP-Mod (select), **B** MCP-Mod (average), **C** FP1, **D** FP2, **E** LS2m, **F** LS2e

Estimated duration-response curves from the model-based fits to the data generated by the logistic data-generating mechanism (solid black line) with a sample size of 30 participants per duration (150 total) are shown in Fig. 3. The parameterizations of the linear spline models appear to force an over-complicated estimated shape. The fractional polynomial models tend to fare better, though FP2’s tendency to under- and overestimate the response rates corresponding to lower durations is concerning. Of particular concern is the overestimation of the response rate at low durations, which could lead to falsely estimating a MED considerably lower than the true MED. The MCP-Mod methods both appear to generally mimic the proper shape, with a slightly lower, though non-negligible risk of overestimation at lower durations and occasional risk of incorrectly suggesting a non-monotonic relationship.

**Fig. 4:**
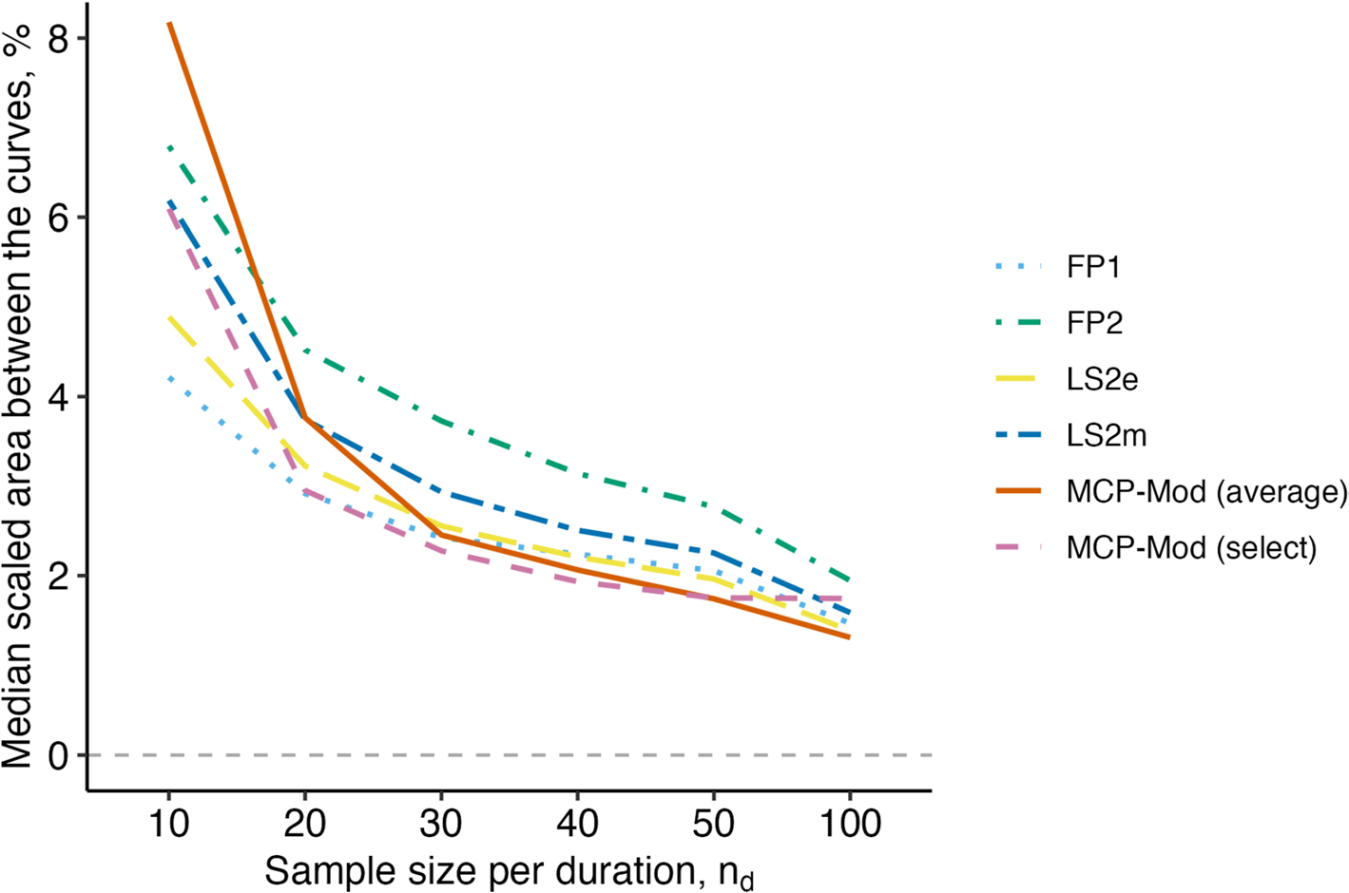
The median sABC (%) from the estimated fits from 5,000 simulated datasets for each method, by sample size assigned to each duration ($n_d$). The data were simulated based on the logistic data-generating mechanism. A sABC (%) of zero would indicate a perfect fit

To quantify model-based performance in estimating the duration-response curves, we examine the results of the sABC analysis (Fig. 4). Only the results for the logistic data-generating mechanism are shown here, though results across all data-generating mechanisms were similar (Fig. S3). The sABC metric captures the average absolute error in estimating the probability of cure across the whole duration range, reported here as a percentage. Figure 4 shows the median average absolute error (%) for each method across the 5000 simulated datasets. The results from this analysis reinforce what was observed in Fig. 3. All methods are quite accurate at large sample sizes, with a maximum average absolute error of less than 2.5% when 100 participants are assigned to each duration ($n_{total}=500$). As sample size decreases, FP2 tends to have the highest error in estimating the probability of cure across the whole range of durations considered. FP1, on the other hand, is consistently competitive with the MCP-Mod approaches in terms of lowest average absolute error.

**Fig. 5:**
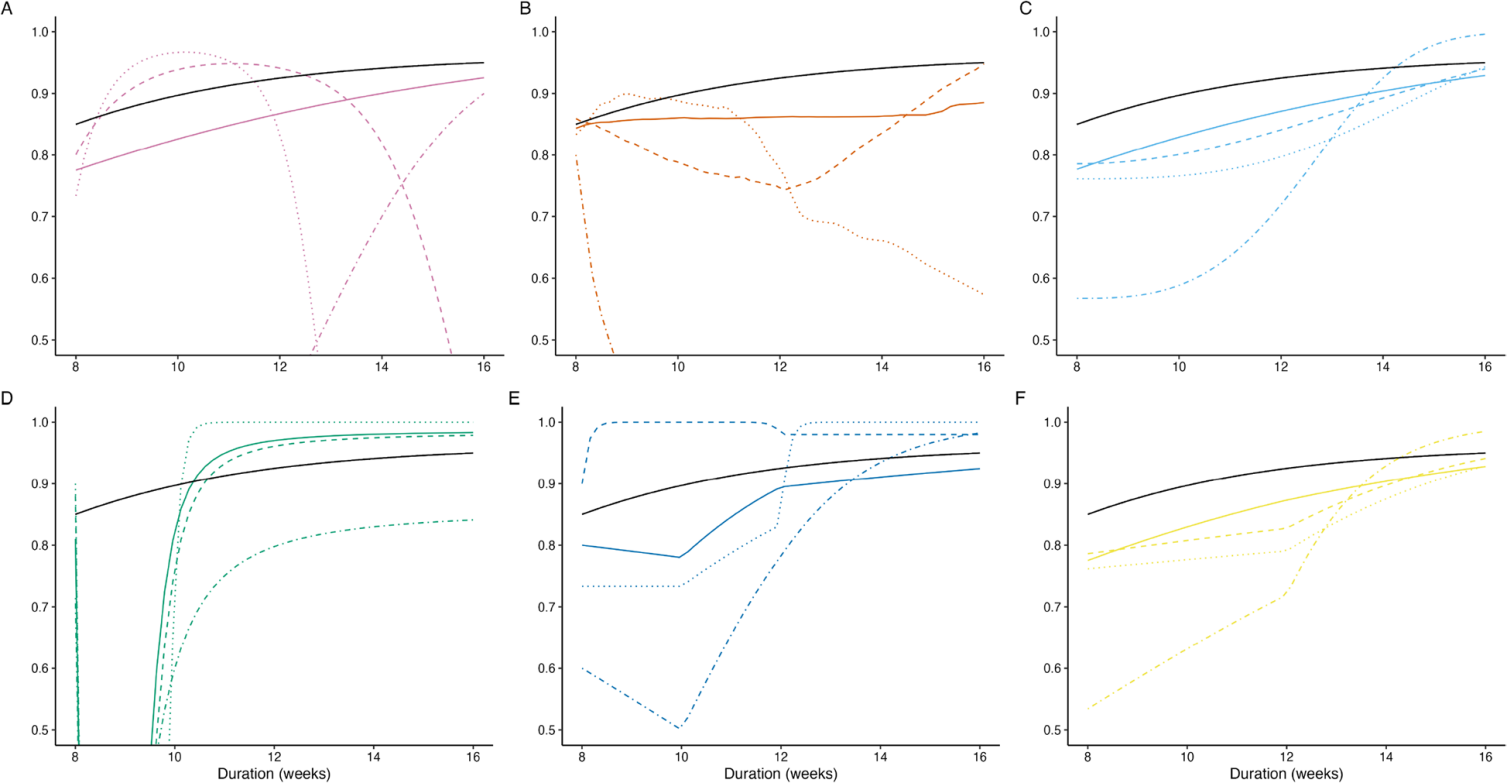
The estimated duration-response curves corresponding to the largest (worst) sABC for sample sizes of 10 (dot-dashed), 30 (dotted), 50 (dashed), and 100 (solid) per duration when the true data-generating distribution is one of logistic growth (solid black line). **A** MCP-Mod (select), **B** MCP-Mod (average), **C** FP1, **D** FP2, **E** LS2m, **F** LS2e

Figure 5 shows the estimated duration-response curves associated with the worst model fits (largest sABC) for sample sizes of 10, 30, 50, and 100 per duration. FP2 (Fig. 5D) and LS2m (Fig. 5E) have erratic worst fits that overestimate the effectiveness associated with shorter durations. FP1 (Fig. 5C) and LS2e (Fig. 5F) have somewhat desirable worst fits in terms of monotonic shapes and conservative estimation of the duration-response relationship. Finally, the differences in the MCP-Mod (select) and MCP-Mod (average) methods (Fig. 5A, B) are well illustrated here. When using MCP-Mod (select) with a small sample size, there is a risk of performing estimation based on an incorrect model. This risk is mitigated when MCP-Mod averages across best model fits. Further performance improvement may be possible with an increased number of bootstrap samples.

### Performance: estimation of optimal duration

Table 4 displays the true MED for each data-generating mechanism alongside the median estimated MED from applying each method to the 5000 simulated datasets per data-generating mechanism with 30 participants per duration ($n_{total} = 250$). All model-based methods, with the notable exception of MCP-Mod (average), display a tendency to underestimate the MED at this sample size. Careful consideration of acceptable error margins, or the use of a more conservative boundary such as the duration at which the estimated lower confidence bound exceeds 90%, will be critical in mitigating risk. In stark contrast, the Dunnett Test approach consistently failed to identify a MED shorter than the longest duration observed. Even at 100 participants per duration, 82.2% of Dunnett estimates suggested 16 weeks as the optimal duration (Supplemental Figure S2).

**Table 4:** The true minimum effective duration (MED) in weeks for each data-generating mechanism and the median (IQR) estimated MED from each method when applied across 5,000 simulated datasets with 30 participants per duration

	Logistic	Linear in Log-Odds	Linear
True MED	10.2	11.1	12.0
Estimated MED (IQR)			
FP1	9.5 (8.2, 11.6)	10.6 (8.2, 12.4)	11.4 (8.3, 12.9)
FP2	9.6 (8.2, 10.9)	10.1 (8.2, 11.8)	10.6 (8.2, 12.2)
LS2e	10.0 (8.7, 11.3)	10.4 (8.7, 12.1)	10.9 (8.7, 12.7)
LS2m	9.3 (8.2, 10.8)	10.1 (8.2, 11.6)	10.1 (8.0, 12.1)
MCP-Mod (average)	10.3 (8.3, 12.4)	11.1 (8.7, 13.2)	11.8 (8.5, 13.7)
MCP-Mod (select)	9.8 (8.3, 11.6)	10.6 (8.5, 12.2)	11.1 (8.8,12.9)
Dunnett Test (contiguous)	14.0 (12.0, 14.0)	14.0 (12.0, 14.0)	14.0 (14.0, 14.0)
Dunnett Test (absolute)	12.0 (10.0, 14.0)	12.0 (10.0, 14.0)	12.0 (10.0, 14.0)

Dunnett Test only shows results when the MED was estimated to be below 16 weeks (<5% of results)

To further understand estimator robustness to sample size, we examine the range in estimated MEDs across the simulated datasets. For most model-based methods the estimated MED IQR spans nearly 3-weeks, with 25% of the simulated data results returning estimated MEDs at or below 8 weeks. This behavior does improve as sample size increases (Supplemental Material, Table S3) and will benefit from the application of a more conservative boundary (e.g., using the duration whose lower confidence bound exceeds 90%).

**Fig. 6:**
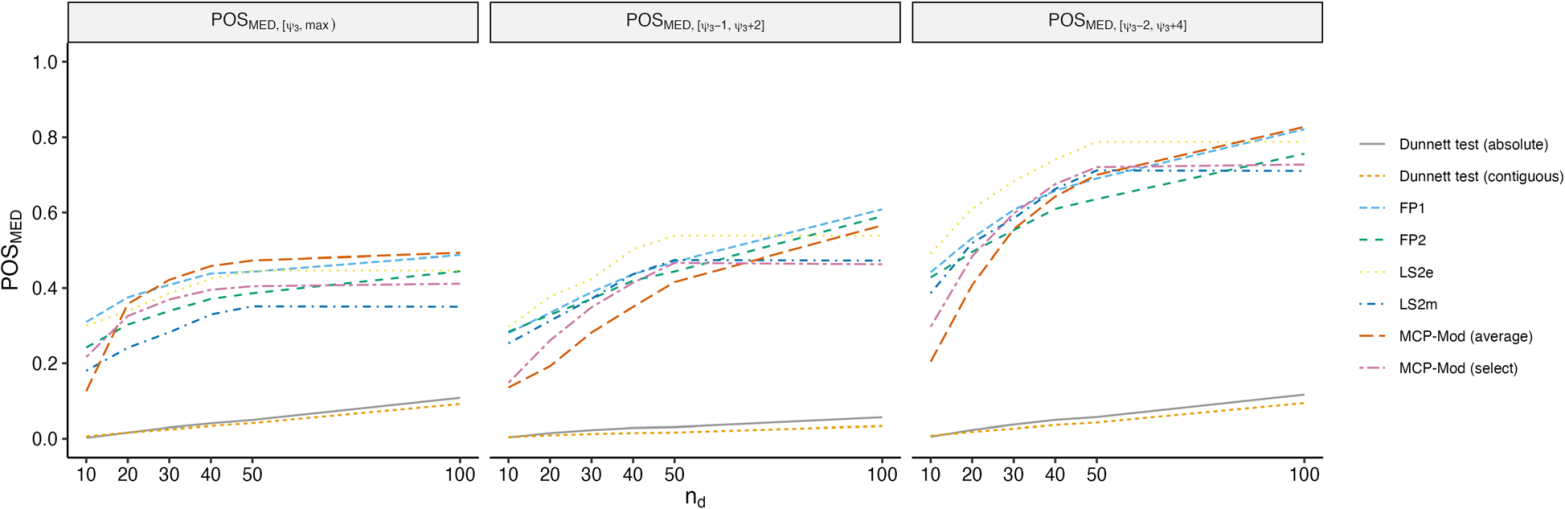
Estimated $\text {POS}_{\text {MED}}$ for **A** $\text {POS}_{\text {MED}, [0,\max )}$, **B** $\text {POS}_{\text {MED}, [-1,+2]}$, and **C** $\text {POS}_{\text {MED}, [-2,+4]}$ across 15,000 simulated datasets (5000 simulated datasets for each DGM)

The $POS_{\text {MED}}$ performance results are shown in Fig. 6. The improvement in $\text {POS}_{\text {MED}}$ performance across the three panels appears to be due to increasing tolerance of slight underestimation of the MED. The first panel (Fig. 6: $\text {POS}_{\text {MED},[\psi _3,max]}$) appears to contain the lowest proportion of acceptable estimates. The second panel (Fig. 6: $\text {POS}_{\text {MED},[\psi _3-1,\psi _3+2]}$) returns improved performance results despite enforcing a far stricter interval. The only method whose performance appears worse when comparing these two panels is the MCP-Mod (average) method. The last panel (Fig. 6: $\text {POS}_{\text {MED},[\psi _3-2,\psi _3+4]}$) has an upper bound on the acceptable region of estimated MEDs that is similar to the “acceptable power” interval. However, the performance is strikingly improved relative to the first panel, reflecting the tendency towards underestimation that falls into the acceptable interval for $\text {POS}_{\text {MED},[\psi _3-2,\psi _3+4]}$ but outside of the acceptable interval for $\text {POS}_{\text {MED},[\psi _3,max)}$.

## Discussion

In this work we have adapted popular model-based dose-ranging techniques, including MCP-Mod and fractional polynomials, for the purposes of duration-ranging. We have shown through simulation that such model-based methods outperform standard qualitative comparisons on every target, particularly in the constrained sample sizes of a Phase II trial. Though no method, model-based or qualitative, achieved high power at the lowest sample sizes for the simulation settings examined, FP1 dominated all other approaches. With respect to accurately reproducing the true duration-response curve, MCP-Mod and FP1 had the lowest average error. This task is not feasible with qualitative methods and is a direct advantage of implementing model-based duration estimation strategies. The linear splines were relatively unreliable in replicating the duration-response curve, with knot specification playing an overly influential role. Finally, as in the dosing literature, we observed the tendency to overestimate the optimal duration when standard qualitative comparisons were implemented for analysis. This effect is due in part to the inability to directly target the desired MED and instead having to apply a conservative testing framework. Model-based methods were more agile in identifying the MED, but did tend to underestimate. This behavior can be mitigated through the application of a more conservative threshold, such as using a lower confidence bound to identify the MED. FP2 appears to be particularly susceptible to underestimation of the MED at smaller sample sizes and if a confidence bound-based threshold is implemented, the FP models must account for model-selection to guard against further anti-conservative estimation. This is further described by Pham et al. [29]. In summary, MCP-Mod and FP1 are reliable approaches for future Phase II trials targeting the estimation of the MED, with MCP-Mod and model-averaging at a slight advantage when more complex duration-response relationships are expected.

Power to detect a non-flat duration-response relationship was quite low across the simulation settings and methods. This is due in part to the narrow range of expected effect sizes across the simulation study, but is also worth further investigation in this particular context. In placebo-controlled dose-ranging, evidence of effect ($\psi _1$) must typically be established in order to determine the optimal dose. In the duration-ranging setting where a known effective duration serves as the maximum duration under evaluation, establishment of a duration-response relationship is not a requirement and, in fact, its absence may be desirable in that it suggests the maximum duration may not be the optimal duration. In this sense, estimating the optimal duration is philosophically distinct from estimating optimal dose; hence, estimating the MED ($\psi _3$) is not dependent on establishing evidence of effect ($\psi _1$).

This work did not examine the impact of design parameters on performance, such as the number of durations observed, the spacing between durations, nor the (im)balance of sample size across durations. The work by [4] suggests that reasonable coverage of the duration-response curve, even if durations are non-equidistant and sample size is somewhat imbalanced, should result in similar results though, naturally, be subject to error when unanticipated non-linearity of the curve is not observed.

Future work is underway to incorporate patient characteristics and risk profiles into both the data-generating mechanism and the method of analysis. Information on the inclusion of covariates for the MCP-Mod procedure is available, but limited. Further development will be needed to inform the inclusion of covariate information in a duration-response setting. This work also does not address the setting where the true MED falls outside of the observation region, either at a lower duration than randomized to or, in a less likely scenario, at a higher duration than the maximum observed.

At the 2014 workshop on dose finding hosted by the EMA and European Federation of Pharmaceutical Industries and Associations, the gathered experts came to the clear and compelling agreement that “selection of dose for phase III is an estimation problem and should not be addressed via hypothesis testing” [30]. Duration ranging should equally receive such directional reframing. Randomized trial design must evolve to hone the ability to directly estimate the optimal duration. In this regard, designs such as MAMS-ROCI [22] and TB therapeutics trials such as SPECTRA-TB (ACTG A5414) and DRAMATIC (NCT03828201) lead the way toward a new era of treatment shortening efforts.

## Supplementary Information

Supplementary Material 1.

## Data Availability

All code necessary for data generation, analysis, and report compilation is publicly available in a GitHub repository maintained by the first author (https://github.com/sdufault15/tb-mcp-mod).

## Abbreviations

TB: Tuberculosis
MCP-Mod: Multiple comparison procedure and modeling approach
MAMS-ROCI: Multi-Arm Multi-Stage Response Over Continuous Intervention design
REMoxTB: Rapid Evaluation of Moxifloxacin in the Treatment of Sputum Smear Positive Tuberculosis Trial
WHO: World Health Organization
ANOVA: Analysis of variance
FDA: United States Food and Drug Administration
MCP: Multiple comparison procedures
Mod: Modeling procedure
ADEMP: Aims, Data-generating mechanisms, Methods, Estimands, Performance Measures framework
DS-TB: Drug-sensitive tuberculosis
MED: Minimum effective duration
PoC: Proof of concept
FP1: Fixed 1-degree fractional polynomial
FP2: Fixed 2-degree fractional polynomial
LS2e: Piece-wise linear spline with 2 equally spaced knots
LS2m: Piece-wise linear spline with 2 manually placed knots
sABC: Scaled area between the curves
POS_MED_: Probability of successfully estimating the minimum effective duration within a pre-specified interval around the truth
DGM: Data-generating mechanism
IQR: Interquartile range

## References

[1] World Health Organization. Global Tuberculosis Report 2023. Geneva: WHO; 2023.

[2] NIAID Tuberculosis Research Strategic Plan Working Group. NIAID Strategic Plan for Tuberculosis Research. 2019.

[3] Imperial MZ, Phillips PJ, Nahid P, Savic RM. Precision-enhancing risk stratification tools for selecting optimal treatment durations in tuberculosis clinical trials. Am J Respir Crit Care Med. 2021;204(9):1086–96. 34346856 10.1164/rccm.202101-0117OC PMC8663006

[4] Quartagno M, Walker AS, Carpenter JR, Phillips PPJ, Parmar MKB. Rethinking non-inferiority: a practical trial design for optimising treatment duration. Clin Trials. 2018;15(5):477–88. 29871495 10.1177/1740774518778027 PMC6136078

[5] Working Group for New TB Drugs. Clinical pipeline [Internet]. 2024 [cited 2025 Apr 14]. https://www.newtbdrugs.org/pipeline/clinical. Accessed 2 July 2024.

[6] Horsburgh CR, Shea KM, Phillips P, LaValley M. Randomized clinical trials to identify optimal antibiotic treatment duration. Trials. 2013;14(1): 88. 23536969 10.1186/1745-6215-14-88 PMC3622584

[7] Gillespie SH, Crook AM, McHugh TD, et al. Four-month moxifloxacin-based regimens for drug-sensitive tuberculosis. N Engl J Med. 2014;371(17):1577–87. 25196020 10.1056/NEJMoa1407426 PMC4277680

[8] Li S-Y, Irwin SM, Converse PJ, et al. Evaluation of moxifloxacin-containing regimens in pathologically distinct murine tuberculosis models. Antimicrob Agents Chemother. 2015;59(7):4026–30. 25918146 10.1128/AAC.00105-15 PMC4468727

[9] Diacon AH, Pym A, Grobusch MP, et al. Multidrug-resistant tuberculosis and culture conversion with bedaquiline. N Engl J Med. 2014;371(8):723–32. 25140958 10.1056/NEJMoa1313865

[10] World Health Organization. WHO consolidated guidelines on tuberculosis. Module 4: treatment-drug-resistant tuberculosis treatment, 2022 update. 2022. 36630546

[11] Bretz F, Hsu J, Pinheiro J, Liu Y. Dose finding–a challenge in statistics. Biom J. 2008;50(4):480–504. 18663758 10.1002/bimj.200810438

[12] Schorning K, Bornkamp B, Bretz F, Dette H. Model selection versus model averaging in dose finding studies. Stat Med. 2016;35(22):4021–40. 27226147 10.1002/sim.6991

[13] Bornkamp B, Bretz F, Dmitrienko A, et al. Innovative approaches for designing and analyzing adaptive dose-ranging trials. J Biopharm Stat. 2007;17(6):965–95. 18027208 10.1080/10543400701643848

[14] Thomas N. Hypothesis testing and Bayesian estimation using a sigmoid Emax model applied to sparse dose-response designs. J Biopharm Stat. 2006;16(5):657–77. 17037264 10.1080/10543400600860469

[15] Bretz F, Pinheiro JC, Branson M. Combining multiple comparisons and modeling techniques in dose-response studies. Biometrics. 2005;61(3):738–48. 16135025 10.1111/j.1541-0420.2005.00344.x

[16] USA Food and Drug Administration. Statistical review and evaluation qualification of statistical approach: MCP-Mod. 2016.

[17] European Medicines Agency. Qualification opinion of MCP-Mod as an efficient statistical methodology for model-based design and analysis of Phase II dose finding studies under model uncertainty. 2014.

[18] Morris TP, White IR, Crowther MJ. Using simulation studies to evaluate statistical methods. Stat Med. 2019;38(11):2074–102. 30652356 10.1002/sim.8086 PMC6492164

[19] Bornkamp B, Pinheiro J, Bretz F. MCPmod: an R package for the design and analysis of dose-finding studies. J Stat Softw. 2009;29(7):1–23.

[20] Pinheiro J, Bornkamp B, Glimm E, Bretz F. Model-based dose finding under model uncertainty using general parametric models. Stat Med. 2014;33(10):1646–61. 24302486 10.1002/sim.6052

[21] Liu F, Zhao Q, Rodgers AJ, Mehrotra DV. Calculation of phase 2 dose-finding study sample size for reliable phase 3 dose selection. Pharm Stat. 2023. 10.1002/pst.2330. 37550963 10.1002/pst.2330

[22] Quartagno M, Carpenter JR, Walker AS, Clements M, Parmar MKB. The DURATIONS randomised trial design: estimation targets, analysis methods and operating characteristics. Clin Trials. 2020;17(6):644–53. 33153304 10.1177/1740774520944377 PMC7851720

[23] Bornkamp B, Pinheiro J, Bretz F, Sandig L. DoseFinding: Planning and Analyzing Dose Finding Experiments [Internet]. Version 1.0-2. 2021 [cited 2025 Apr 14]. https://CRAN.R-project.org/package=DoseFinding.

[24] Bojanowski M. lspline: Linear Splines with Convenient Parametrisations [Internet]. Version 1.0-0. 2017 [cited 2025 Apr 14]. https://CRAN.R-project.org/package=lspline.

[25] Rigby RA, Stasinopoulos DM. Generalized additive models for location, scale and shape (with discussion). Appl Stat. 2005;54(3):507–54.

[26] Signorell A. DescTools: Tools for Descriptive Statistics [Internet]. Version 0.99.50. 2023 [cited 2025 Apr 14]. https://CRAN.R-project.org/package=DescTools.

[27] Wickham H, Averick M, Bryan J, et al. Welcome to the tidyverse. J Open Source Softw. 2019;4(43): 1686. 10.21105/joss.01686.

[28] Pedersen TL. patchwork: The Composer of Plots [Internet]. Version 1.1.1. 2020 [cited 2025 Apr 14]. https://CRAN.R-project.org/package=patchwork.

[29] Pham TM, Crook AM, Rolfe K, Phillips PPJ, Dufault SM, Quartagno M. Designing a response-over-continuous-interventions (ROCI) randomised trial: Implementation in the phase 2C part (duration ranging) of the PARADIGM4TB trial. Contemporary Clinical Trials. Elsevier BV; 2025;108002. https://doi.org/10.1016/j.cct.2025.108002. 10.1016/j.cct.2025.108002 PMC13230843 40645367

[30] Musuamba FT, Manolis E, Holford N, et al. Advanced methods for dose and regimen finding during drug development: summary of the EMA/EFPIA workshop on dose finding (London 4–5 December 2014). CPT Pharmacometrics Syst Pharmacol. 2017;6(7):418–29. 28722322 10.1002/psp4.12196 PMC5529745

