# Supplementary material for: Comparison of methods for identifying the optimal treatment duration in randomized trials for antibiotics
Source: Trials. 2025 Sep 24;26:352. doi: 10.1186/s13063-025-09050-y (PMC12462354; doi:10.1186/s13063-025-09050-y)
Supplement: Supplementary file 1 — Supplementary Material 1. [file 13063_2025_9050_MOESM1_ESM.pdf]

COMPARISON OF METHODS FOR IDENTIFYING THE OPTIMAL TREATMENT DURATION IN RANDOMIZED TRIALS FOR ANTIBIOTICS

A PREPRINT

Suzanne M. Dufault*
Division of Biostatistics
Department of Epidemiology and Biostatistics
University of California, San Francisco
San Francisco, CA
suzanne.dufault@ucsf.edu

Brian H. Aldana
Division of Pulmonary and Critical Care Medicine
Department of Medicine
University of California, San Francisco
San Francisco, CA
brian.aldana@ucsf.edu

Patrick P.J. Phillips
Division of Pulmonary and Critical Care Medicine
Department of Medicine
University of California, San Francisco
San Francisco, CA
patrick.phillips@ucsf.edu

April 9, 2025

Supplemental Material

S1 Data-Generating Mechanisms

*Corresponding author

**Model Name**	**Parameterization,** $\mathbb{E}[Y\|X=x]$	**True MED (weeks)**
Logistic	$\frac{0.961}{1+1.468\exp(-0.302x)}$	10.2
Linear in log-odds	$\frac{1}{1+\exp\{-(\text{logit}(0.85)+\frac{\text{logit}(0.95)-\text{logit}(0.85)}{8}(x-8)\}}$	11.1
Linear	$0.75+0.0125x$	12.0

Table S1: Parameterizations of the data-generating mechanisms used for the simulation study and the corresponding minimum effective duration (MED) associated with a 90% response rate.

S2 Methods of Analysis: Parameterizations

MCP-Mod.

Model	Standardized Functional Form, $f^0(d,\theta^0)$	Model Specifications
EMax	$d/(ED_{50}+d)$	$E_0=0.85, ED_{50}=2$
EMax	$d/(ED_{50}+d)$	$E_0=0.85, ED_{50}=6$
Sigmoid Emax	$d^h/(ED_{50}^h+d^h)$	$E_0=0.85, ED_{50}=8, h=3.5$
Quadratic	$d+\delta d^2$	$\delta=\beta_2/\|\beta_1\|=-0.0776$
Linear	d	$\beta_0=0.85, \beta_1=0.1/8$

Table S2: The models included in the MCP-Mod candidate library and their initial specifications.

Where $f(d,\theta)=\theta_0+\theta_1 f^0(d,\theta^0)$. See Bornkamp et al (2009) for more details.

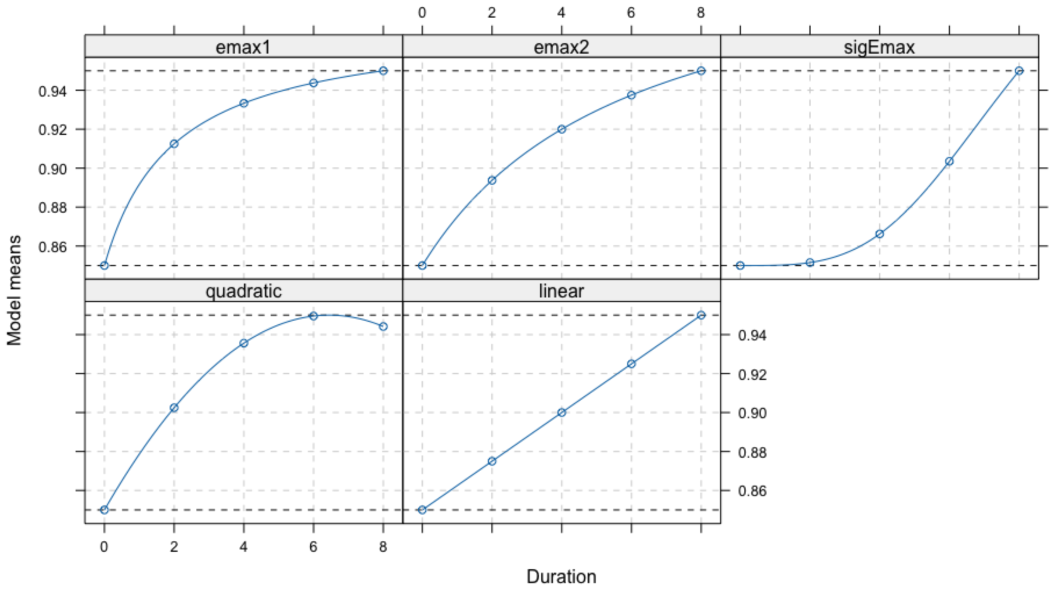

Figure S1: The shapes included in the MCP-Mod candidate library.

Fractional Polynomials.

$$\mathbb{E}[Y|X=x] = \beta_1 x^{p_1} \quad \text{FP1} \tag{1}$$

$$\mathbb{E}[Y|X=x] = \beta_1 x^{p_1} + \beta_2 x^{p_2} \quad \text{FP2} \tag{2}$$

$$\tag{3}$$

Where the powers p_1, p_2 are taken from the set $S = \{-2, -1, -0.5, 0, 0.5, 1, 2, 3\}$ and selected by the internal `gamlss` algorithm based on best fit for each simulated dataset.

Linear splines.

$$\mathbb{E}[Y|X=x] = s_0 + s_1\mathbb{I}\{x < 10.667\}d + s_2\mathbb{I}\{x \in [10, 13.333)\}d + s_3\mathbb{I}\{x \geq 13.333\}d \quad \text{LS2e} \tag{4}$$

$$\mathbb{E}[Y|X=x] = s_0 + s_1\mathbb{I}\{x < 10\}d + s_2\mathbb{I}\{x \in [10, 12)\}d + s_3\mathbb{I}\{x \geq 12\}d \quad \text{LS2m} \tag{5}$$

S3 Additional Results

	Logistic	Linear in Log-Odds	Linear
True MED	10.2	11.1	12.0
LS2e	12.1 (10.8, 12.9)	10.9 (10.1, 12.2)	10.3 (9.6, 10.9)
LS2m	11.4 (10, 12.7)	10.8 (9.5, 11.8)	10.1 (9.3, 10.9)
MCP-Mod (average)	11.9 (10.8, 12.9)	11.1 (9.8, 12.1)	10.1 (9.1, 11.1)
MCP-Mod (select)	11.4 (9.6, 12.9)	10.6 (9, 12.1)	10 (8.7, 11.3)
FP1	12.1 (10.8, 12.9)	11.1 (9.6, 12.1)	10 (8.7, 11.1)
FP2	11.6 (10.4, 12.7)	10.8 (9.3, 11.8)	10.1 (8.2, 10.9)
Dunnett (absolute)	14 (14, 14)	14 (12, 14)	14 (12, 14)
Dunnett (contiguous)	14 (14, 14)	14 (14, 14)	14 (12, 14)

Table S3: The true minimum effective duration (MED) in weeks for each data-generating mechanism and the median (IQR) estimated MED from each method when applied across 5,000 simulated datasets with 100 participants per duration.

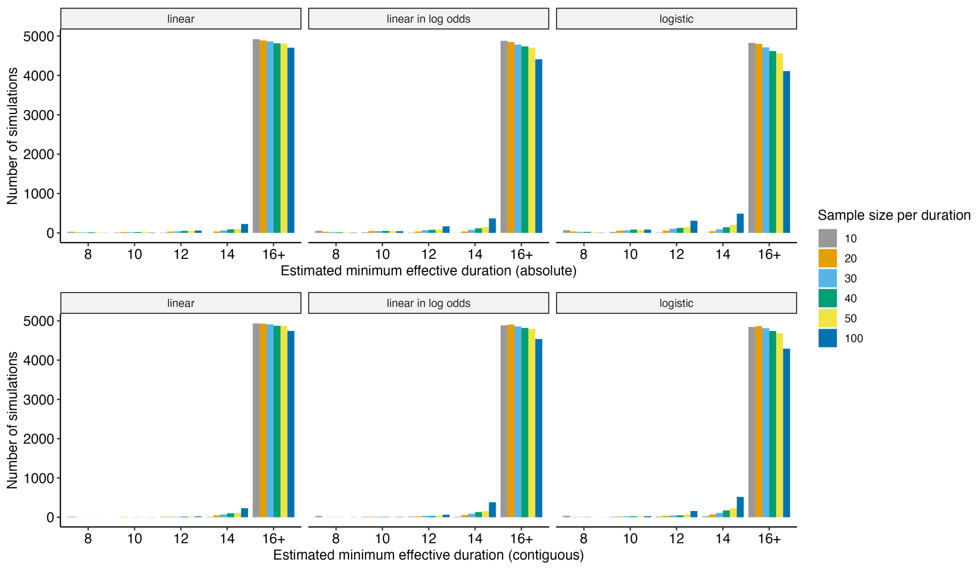

Figure S2: The estimated minimum effective durations from applying the Dunnett Test to 5,000 simulated datasets at each sample size.

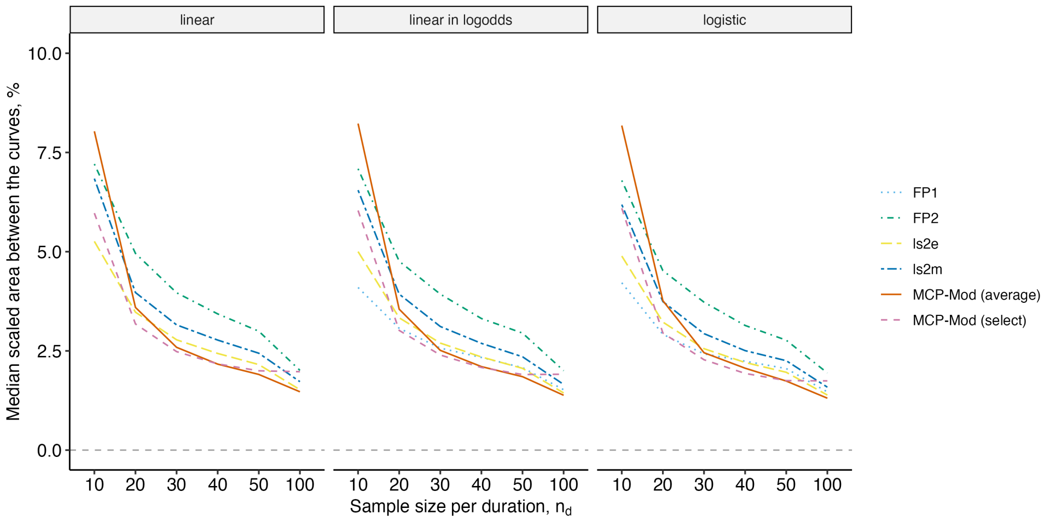

Figure S3: The median scaled area between the curves (%) from the estimated fits from 5,000 simulated datasets of each sample size (n_d) for each data-generating mechanism and modeling method. A scaled area between the curves (%) of zero would indicate perfect fit.

References

Bornkamp B, Pinheiro J, Bretz F. MCPMod: An R Package for the Design and Analysis of Dose-Finding Studies. Journal of Statistical Software. 2009 Feb;29(7).
